# The Registration of Nurses

**Published:** 1887-10-29

**Authors:** 


### THE REGISTRATION OF NURSES.

The rules for the register of trained nurses have been printed, and will be forwarded, with form of application, to any nurse sending a stamped and addressed envelope to Mr. Howard J. Collins, Secretary, The Hospitals Association, Norfolk House, Norfolk Street, W.C.

1. A nurse seeking to be placed on the register must furnish satisfactory proof that she has worked for at least one year on the staff of a hospital or infirmary, and that she has been trained in the duties of a nurse. She must also bring a certificate, or testimonial, of good moral and general conduct from the matron, or lady superintendent, of the hospital or infirmary in which she has been trained.

2. An entrance fee of 2s. 6d., which covers cost of the Association's badge, and an annual subscription of one shilling, will be charged to each nurse.

3. A nurse will forfeit her badge for intemperance or other serious misconduct, or neglect of professional duties.

4. Nurses making application to be placed on the register must first communicate with the secretary by letter addressed to the office of The Hospitals Association, Norfolk House, Norfolk Street, London, W.C.

*** Dr. Steele, Superintendent of Guy's Hospital, will read a paper on the "Registration of Nurses," in the Court Room of Guy's Hospital, St. Thomas Street, S.E. (London Bridge Station), at 8 p.m. on Wednesday, December 14th next, when Sir Andrew Clark, Bart., M.D., will preside.

